# The Effect of Oxalic Acid as the Pre-Activator for the Electropolishing of Additive Manufactured Titanium-Based Materials and Its Characterization

**DOI:** 10.3390/polym14194198

**Published:** 2022-10-06

**Authors:** Chun-Hao Chen, Chia-Yu Lee, Ming-Der Ger, Shun-Yi Jian, Jung-Chou Hung, Po-Jen Yang, Chun-Hsiang Kao, Yi-Cherng Ferng, Ying-Sun Huang, Kuo-Kuang Jen

**Affiliations:** 1 Fraunhofer Institute für Zuverlässigkeit und Microintegration, IZM, 13355 Berlin, Germany; 2 Graduate School of Defense Science, Chung Cheng Institute of Technology, National Defense University, Taoyuan 33551, Taiwan; 3 Department of Chemical & Materials Engineering, Chung Cheng Institute of Technology, National Defense University, Dasi District, Taoyuan 33551, Taiwan; 4 System Engineering and Technology Program, National Chiao Tung University, Hsinchu City 300, Taiwan; 5 Department of Material Engineering, Ming Chi University of Technology, New Taipei City 24301, Taiwan; 6 Center for Plasma and Thin Film Technology, Ming Chi University of Technology, New Taipei City 243303, Taiwan; 7 Department of Mechanical Engineering, National Central University, Chung-Li 320, Taiwan; 8 Missile and Rocket Systems Research Division, National Chung-Shan Institute of Science and Technology, Taoyuan 32546, Taiwan

**Keywords:** oxalic acid, electropolishing, additive manufacturing, surface roughness, passive layer

## Abstract

The use of additive manufactured (AM) titanium-based materials has increased substantially for medical implants and aerospace components. However, the inferior surface roughness of additive manufactured products affects the outward appearance and reduces performance. This study determines whether activation treatment prior to electropolishing produces a better surface. Oxalic acid (OA) is used as a pre-activator using different experimental conditions and the surface roughness is reduced by electropolishing with an electrolyte of perchloric acid and glacial acetic acid. The SEM surface morphology, mechanical properties, phase transformation and electrochemical properties are measured to determine the effect of different degrees of roughness on the surface. The results show that the surface roughness of AM titanium-based samples decreases from 8.47 µm to 1.09 µm after activation using OA as a pre-treatment for electropolishing. After electropolishing using optimal parameters, the hardness and resistance to corrosion resistance are increased.

## 1. Introduction

Titanium (Ti) was discovered in 1790 but was not purified until the early 1900s. Titanium was not widely used until the second half of the twentieth century [1]. However, titanium is now widely used in military applications, sports equipment, the aerospace industry, marine engineering, biomaterials for orthopedic replacements and dental implantations. Titanium is well suited to applications in the aerospace industry and it is the most commonly used element because it exhibits superior corrosion resistance, biocompatibility, relatively low modulus, good fatigue strength, formability, machining ability, stable mechanical properties and low specific weight [2,3,4,5,6].

There are more than a hundred types of Ti-based alloys for different applications. Ti-based alloys are also widely used in additive manufacturing (AM). AM or 3D printing is used to construct 3D objects by adding layers of material, such as plastic, metal, ceramics or human tissue. In 2009, the American Society for Testing Materials (ASTM) standardized this technology as AM [7,8].

The penetration of AM equipment is constrained by problems. The immaturity of AM technology also means there are currently few materials that must be standardized. Objects that are fabricated using AM exhibit surface roughness because powder particles stick to the molten surface contour during manufacturing. This roughness causes stress concentration and crack initiation and has a negative impact on the fatigue performance, so roughness is decreased by post-processing [9,10]. Milling and blasting are used to process AM parts but milling cannot be used for complex geometries that involve undercuts or inner structures and blasting is suited to complicated surfaces, but these are mechanical processes that reduce surface roughness through direct physical contact [11,12].

Electropolishing is a surface treatment that polishes the sample (anode) in a concentrated electrolyte. Electropolishing involves an electrolysis reaction, surface reduction and polishing in a concentrated electric field [13,14,15,16,17,18,19,20,21,22,23,24,25]. The concentrated electric field produces the polished surface. The field is concentrated on peaks on the surface and the local electrical density is increased, but the electrical density is less in grooves. The surface becomes flat and polished. The surface morphology changes and increasing the reaction area decreases the time for the workpiece surface to change from a passivated state to an activated state. 

The reactivity of metal ions and metal oxides after pre-treatment is important [26]. The sample is pretreated before the electropolishing process, which removes passive layers and induces a rapid increase in activity and changes the surface properties [27,28]. The reaction is a surface chemical reaction because passive layers hinder electron transfer. Passive layers inhibit surface corrosion and prolong the electropolishing process.

A protective passive layer is produced by the spontaneous oxidation in air on the surface of Ti-based alloys. A corrosion process or a specific pre-treatment to create passive layers decreases activity and longevity and the efficiency of the electropolishing process is increased. Carboxylic acids, including oxalic acid (OA), increase the reactivity by removing passive layers on the surface of a Ti-based alloy [29,30]. Common carboxylic ligands (OA) increase reactivity by dissolving Ti oxides on the surface of a Ti-based alloy. OA forms soluble complexes to remove passive layers. These Ti–ligand complexes degrade the target compound, depending on their redox properties.

This study determines the effect of immersing titanium alloy in oxalic acid on the electropolishing process. It determines the effect of the electropolishing process, which increases the surface quality without direct physical contact. Samples that are fabricated using an AM process are characterized before and after the post process to determine the change in surface morphology. The mechanical properties of different degrees of surface roughness are also discussed.

## 2. Experimental

### 2.1. Materials and Electropolish Parameters

This study uses additive manufactured Ti-based alloy (40 mm × 25 mm × 5 mm) that has a composition of H: 0.012 wt.%, Fe: 0.25 wt.%, O: 0.13 wt.%, C: 0.08 wt.%, N: 0.05 wt.%, Al: 5.5–6.5 wt.%, V: 3.5–4.5 wt.% and a balance of Mg and inductively coupled plasma mass spectrometry (ICP-MS). The activation treatment involves immersing samples in 10 wt.% OA solution for 30 min. Perchloric acid (70% ACS reagent, 100 mL/L) and acetic acid (100% Su-prapur^®^, 900 mL/L) were used to produce the electropolishing solution. A DC power supply with a potential of 25 V was used for all experiments and operation times were 10 min and 20 min. During the experiment, a beaker was placed in ice water bath, in order to maintain a low temperature. The names of the samples for this study are listed in Table 1.

### 2.2. Microstructural Characterization

Scanning electron microscopy (SEM, JEOL JSM-IT100) was used to observe the surface morphology of the sample. Digital 3D white light interferometry (Chroma 7503) was used to scan the sample to determine the surface roughness. The structure of the sample before and after the electropolishing process was examined by X-ray diffraction (XRD, Bruker D2 PHASER). The micro-hardness of each sample was measured using a Vickers micro-hardness device (HVS-1000) with a load of 100 g for 10 s. 

### 2.3. Electrochemical Properties Measurements

The electrochemical performance and corrosion behavior of the specimens were determined using a potentiodynamic polarization test and electrochemical impedance spectroscopy (EIS). A commercial electrochemical analyzer (VERSASTAT4, Princeton Applied Research) and VersaStudio software were used. Various systems combine hardware and VersaStudio software for specific applications. Electrochemical characterization used a three-electrode cell with a saturated calomel electrode (SCE, +0.24 V vs. SHE at 25 °C) as the reference electrode, a platinum plate as the counter electrode and a sample with an area of approximately 1.77 cm^2^ as the working electrode. Further, 3.5 wt.% sodium chloride (NaCl) at 25 °C was used as the media. The polarization curve was measured by sweeping the potential from −200 mV to +500 mV at a scanning rate of 1 mV/s. EIS used a sinusoidal wave with an amplitude of 5 mV in a frequency range of 10^−2^ Hz to 10^5^ Hz. Prior to all electrochemical experiments (the potentiodynamic polarization test and EIS), the samples were immersed in the test solution to achieve a steady open circuit potential (OCP) for about 20 min [31,32,33,34]. 

## 3. Results and Discussion

### 3.1. The Properties of the Pre-Activation Specimens

**Figure 1:**
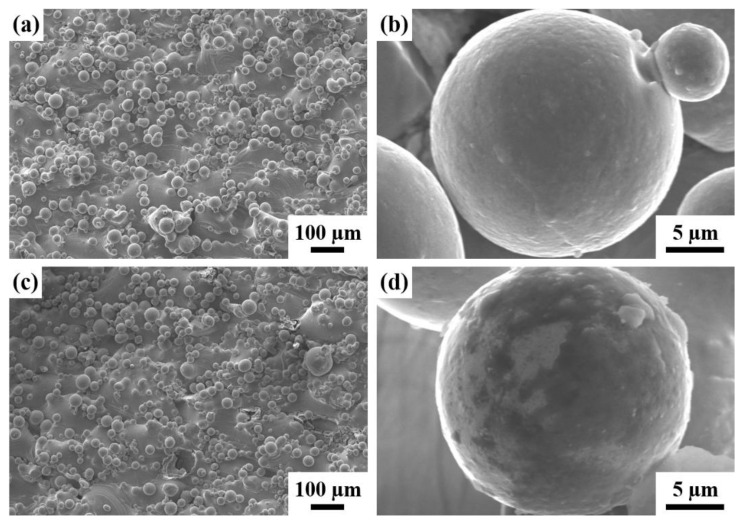
The SEM surface morphology for (**a**,**b**) NAP and (**c**,**d**) AP samples.

The SEM micrographs for NAP and AP samples at magnifications of 100 and 3500 are shown in Figure 1. There is no significant difference between the samples at low magnification, as shown in Figure 1a,c. There is a large quantity of spherical powder bonds all over the surface because there is contact between molten powders and other powders at the interfacial area. The sintered powder forms a molten pool in the molten state instantaneously and adheres to the surrounding powder. These powders bond weakly or fuse incompletely but supersonic vibration does not remove the effect so the additive manufactured sample has high surface roughness. 

Observing a single spheroid powder at high magnification (3500×) shows that there is a difference. As shown in Figure 1b, the NAP sample appears smooth, dense and spherical and there are few satellite powders, which is defined as a larger powder that is attached by one or more than one much smaller powder. Black spots are observed on the surface of the AP sample, where the surface passivation film peels or erodes, as shown in Figure 1d.

**Figure 2:**
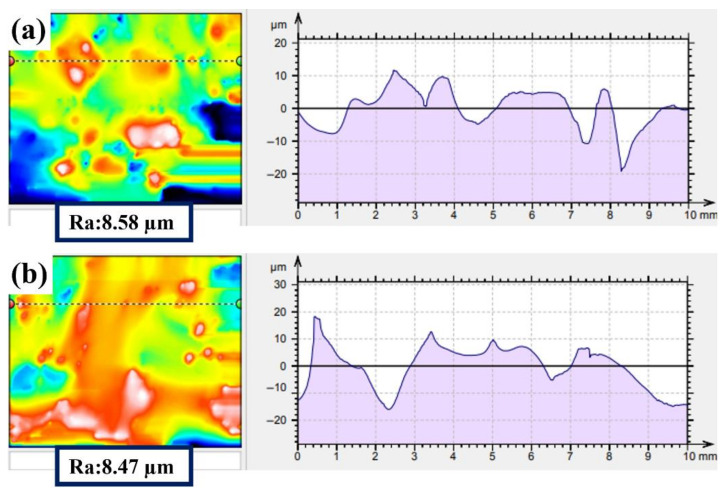
3D white light diagram for (**a**) NAP and (**b**) AP samples.

Figure 2 shows the surface roughness of the NAP and AP samples using 3D white light interferometry, which verifies the results of the SEM surface morphology (Figure 1). The surface roughness of the NAP and AP samples is approximately 8.58 ± 1.14 µm and 8.47 ± 1.11 µm, respectively. This also shows that OA activation does not affect the surface roughness by removing spherical particles: it decreases the effectiveness of the surface passivation film and increases the reaction area. This result is consistent with the SEM surface morphology results (Figure 1).

**Figure 3:**
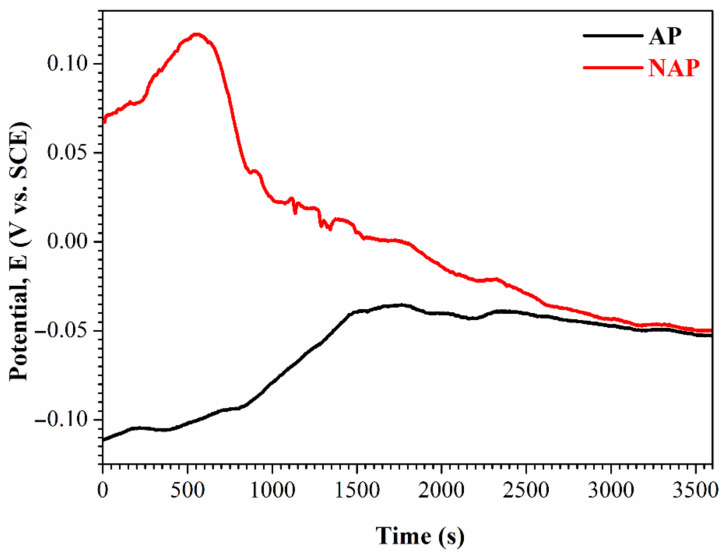
The OCP records of the NAP and AP samples in the electropolishing solution.

To determine whether the surface passivation film peels or erodes, an open circuit potential and potentiodynamic polarization test was performed. Figure 3 shows the OCP results for the NAP and AP samples in the electropolishing solution. The OCP curve gradually becomes smoother as immersion time increases because an oxide passivating film forms. The value of corrosion potential is approximately equal to the OCP reading, which is used to determine the opportunity to initiate a polarization reaction. At the initial stage, the potential value of the AP sample (−0.111 V (vs. SCE)) is much lower than that of the NAP sample (0.067 V (vs. SCE)). This shows that the surface passivation film on the AP sample has defects and poor corrosion resistance. The potential of the AP sample increases as the immersion time increases to 1500 s, at which stage, the surface passivation film grows again. However, the passivation film of the NAP sample remains intact, even in an acidic electropolishing solution, so the potential value begins (at 550 s) to gradually decrease.

**Figure 4:**
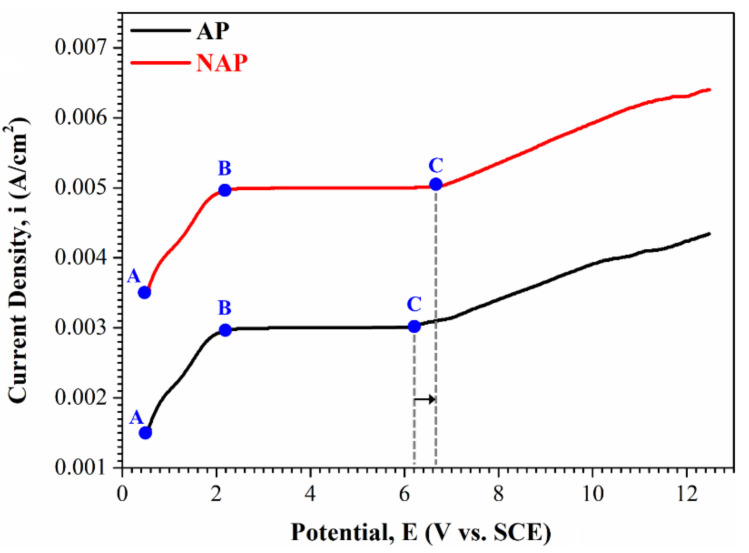
The potentiodynamic polarization curve of the NAP and AP samples electropolishing in the electropolishing solution.

Many studies report that electropolishing is a diffusion-controlled process that occurs at the limiting current [35]. Therefore, the value of the limiting current, which determines the Ti dissolution rate, depends on the rate of mass transfer of Ti ions from the double layer to the bulk of solution. Figure 4 shows the potentialdynamic curve for the electropolishing of the NAP and AP sample in the electropolishing solution. At the initial stage, in the AB region, positive polarization induces an anodic current that increases as the applied potential increases. Dissolution in this region is a charge-transfer-controlled reaction and produces a pitted surface. When the potential increases from point B into the BC region, a viscous layer (passivation effect) begins to form on the anodic surface. In this region, which is called the plateau region, the current is insensitive to the applied potential, so this is the region that is used for the electropolishing process. In the plateau region, a smooth surface is produced because the rate-limiting step is controlled by mass transfer. The passivity electrode/electrolyte layer is crucial for electropolishing, so the passive electrode of the AP sample is at a lower potential than that of the NAP sample, which is consistent with the OCP results. 

### 3.2. The Properties of the Electropolished Specimens

**Figure 5:**
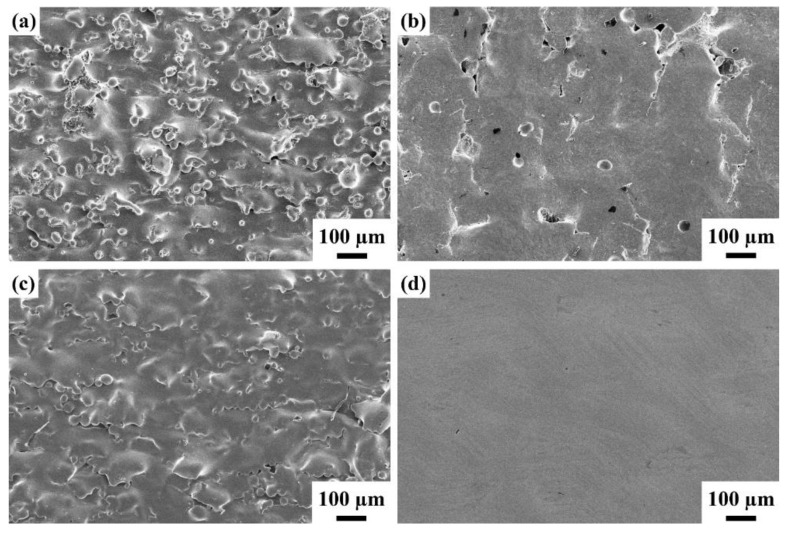
The SEM surface morphology for (**a**) NAPEP10, (**b**) NAPEP20, (**c**) APEP10 and (**d**) APEP20 samples.

Figure 5 shows the surface morphology of various electropolished samples that undergo two different activation treatments (with or without) and electropolishing for a duration of 10 min and 20 min. During electropolishing production, spherical powders attach to the surface, which gradually produces a wave-like morphology for as-fabricated samples with 10 min of electropolishing (Figure 5a,c). The APEP10 sample has a smoother surface than the NAPEP10 sample. The APEP20 sample has the smoothest morphology of all of the electropolished samples, which is a good result. However, there is less bonded powder on the interior surface for samples that do not undergo OA activation and this is virtually eliminated as the duration of electropolishing increases. The SEM images show a wave-like morphology (Figure 5b), which demonstrates the different effects of electropolishing.

**Figure 6:**
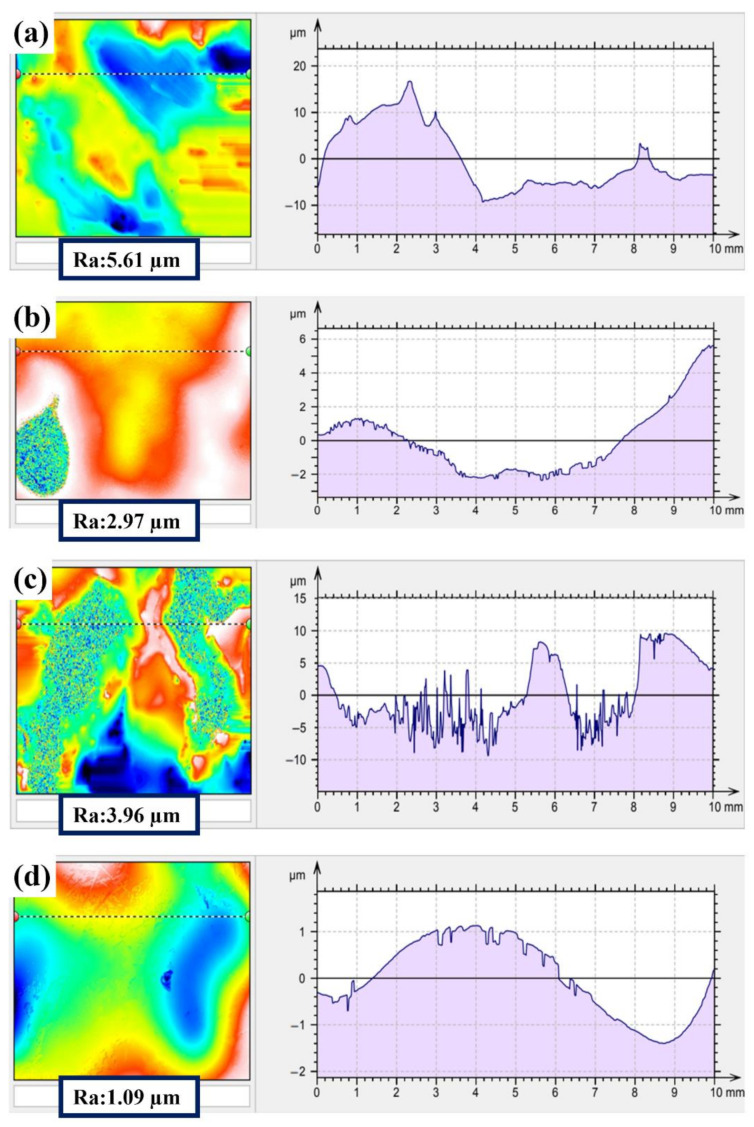
3D white light diagram for (**a**) NAPEP10, (**b**) NAPEP20, (**c**) APEP10 and (**d**) APEP20 samples.

Figure 6 shows the surface roughness measurement results for the same electropolishing process conditions for NAPEP10, NAPEP20, APEP10 and APEP20 samples. The surface roughness of the NAPEP10, NAPEP20, APEP10 and APEP20 samples is approximately 5.61 ± 0.67 µm, 2.97 ± 0.36 µm, 3.96 ± 0.51 µm and 1.09 ± 0.13 µm, respectively. An AP sample that is electropolished for 20 min has the smoothest surface, but the surface roughness of the NAP sample is reduced from about 8.5 µm to approximately 3 µm. OA activation produces a 2 µm difference for the AP sample. The surface roughness of the additive manufactured titanium-based material is reduced from nearly 8.47 µm to nearly 1.09 µm by electropolishing. The respective mass loss (original weight is 19.68 ± 1.29 g) for the AP, NAPEP10, NAPEP20, APEP10 and APEP20 samples is approximately 50 ± 20 mg, 465 ± 70 mg, 895 ± 120 mg, 698 ± 106 mg and 1085 ± 155 mg.

**Figure 7:**
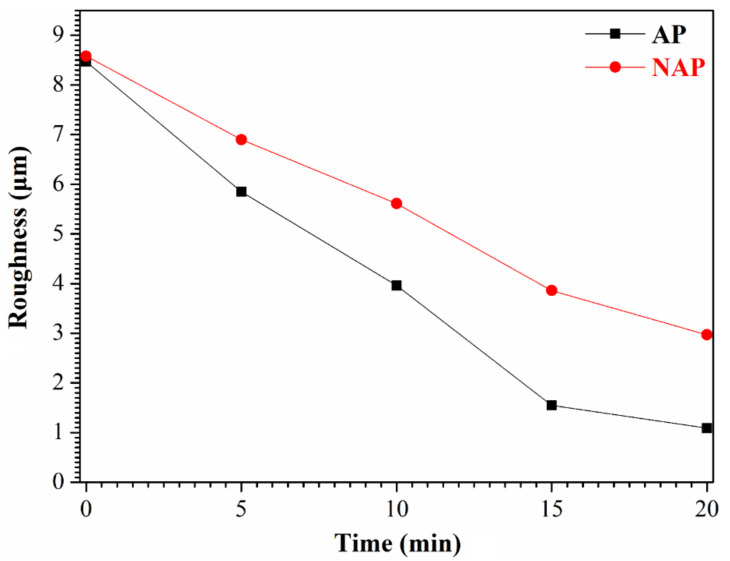
The roughness–time curves of the NAP and AP samples for durations of 5 min, 10 min, 15 min and 20 min.

The electropolishing process uses a constant voltage so duration is the control variable that affects surface roughness. The roughness–time curves for durations of 5 min, 10 min, 15 min and 20 min are shown in Figure 7. The surface roughness of AP samples obviously decreases as operating time increases so OA activation must be used as a pre-treatment for electropolishing.

### 3.3. Comparison of the NAPEP and APEP Samples

AM produces a rough surface due to a melting pool that causes adjacent powder particles that are not melted to be sintered to the surface. The surface quality must be increased by post processing. Electropolishing improves the surface quality of additive manufactured titanium-based materials. This study determines whether an OA activation pre-treatment is effective. It is necessary to determine whether OA activation and the electropolishing affect the basic properties of the material.

**Figure 8:**
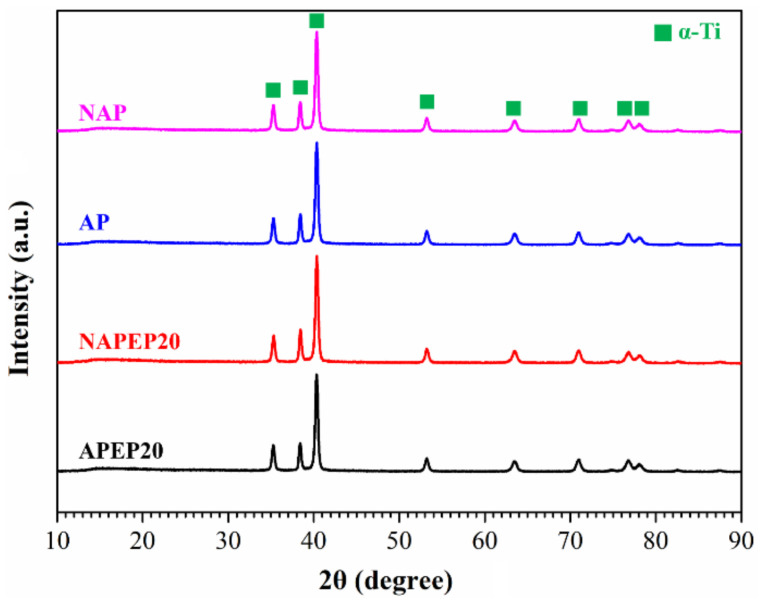
The XRD results of the NAP, AP, NAPEP20 and APEP20 samples.

The surface of the samples undergoes a significant change after OA activation and electropolishing, so it is necessary to confirm whether a phase transformation occurs after these treatments. Figure 8 shows the XRD diffraction patterns for NAP, AP, NAPEP20 and APEP20 samples to confirm the structure of additive manufactured titanium-based materials. The indexing and corresponding Bragg’s peaks that are detected in the XRD scan are attributed to hexagonal close-packed (HCP) α-Ti [36,37]. There is no significant difference in the XRD patterns for NAP and AP samples, whether or not they undergo electropolishing. The presence of α-phase is confirmed at 2θ = 35.33°, 38.6°, 40.47°, 53.29°, 63.72°, 71°, 76.9° and 78.69°. Therefore, it can be concluded that all samples are mostly composed of the α-Ti phase. 

**Figure 9:**
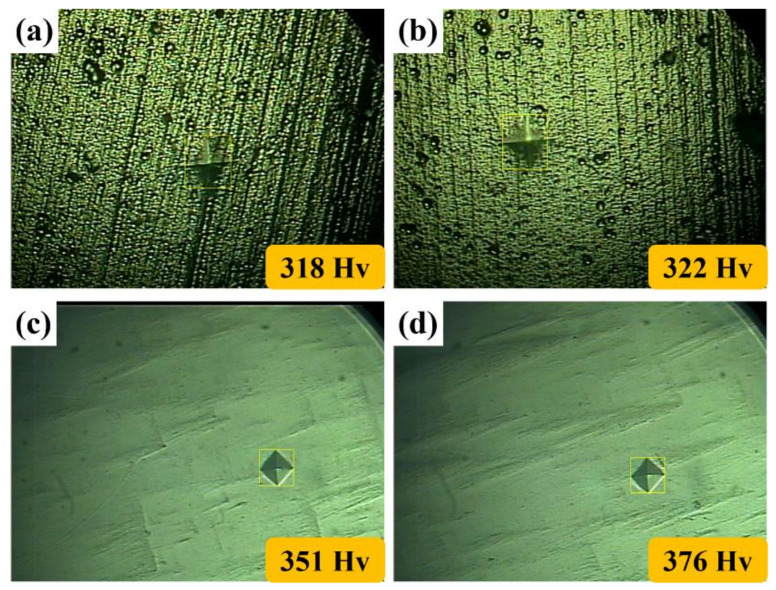
The hardness of (**a**) NAP, (**b**) AP, (**c**) NAPEP20 and (**d**) APEP20 samples.

Figure 9 shows the hardness of NAP, AP, NAPEP20 and APEP20 samples. These are 318 ± 29 HV, 322 ± 30 HV, 351 ± 31 HV and 376 ± 33 HV, respectively. The electropolished samples have higher hardness values than samples that are not electropolished because samples that are not electropolished show crack initiation because stress is concentrated [38,39,40,41].

**Figure 10:**
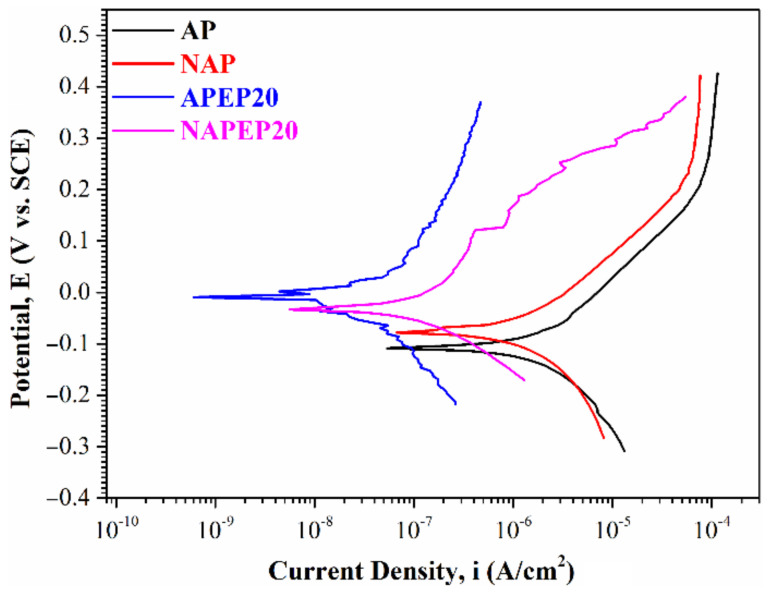
Potentiodynamic polarization curves for the NAP, AP, NAPEP20 and APEP20 samples in 3.5 wt.% NaCl solution.

The potentiodynamic polarization curves for NAP, AP, NAPEP20 and APEP20 samples in 3.5 wt.% NaCl solution are shown in Figure 10. Most of the important corrosion parameters are determined from the curve, including the corrosion potential (*E_corr_*) and the corrosion current density (*i_corr_*). All of the corrosion parameters that are determined using the potentiodynamic polarization curves are listed in Table 2. The *E_corr_* value has a significant effect on the corrosion reaction. A system with a lower *E_corr_* value requires less energy to activate a polarization reaction. 

Figure 10 shows that the corrosion potential for an as-fabricated sample (NAP) is higher than that for an electropolished sample, so the sample that is not electropolished is relatively noble. These results show that the stable oxide layer that forms initially on the sample is removed after electropolishing. When the initial oxide layer is removed, the *E_corr_* value increases as the surface roughness decreases. Therefore, decreasing the surface roughness increases the resistance to polarization. The passive region is decreased after electropolishing. In the passive region, a stable passive layer forms during anodic polarization. The APEP20 sample for Tafel analysis has a broader passive region, so a more protective passive oxide layer is formed. The *i_corr_* value that is determined from the potentiodynamic polarization curve is affected by the corrosion reaction. The results show that the as-fabricated sample (NAP) with the highest surface roughness value features the largest *i_corr_* value and this value decreases during the electropolishing process. An electropolished sample with a lower surface roughness value undergoes a slower corrosion reaction when the passivated oxide film breaks. This occurs if the surface is inhomogeneous because the presence of grooves and crevices with higher energy levels on a rough surface promotes the corrosion reaction.

**Figure 11:**
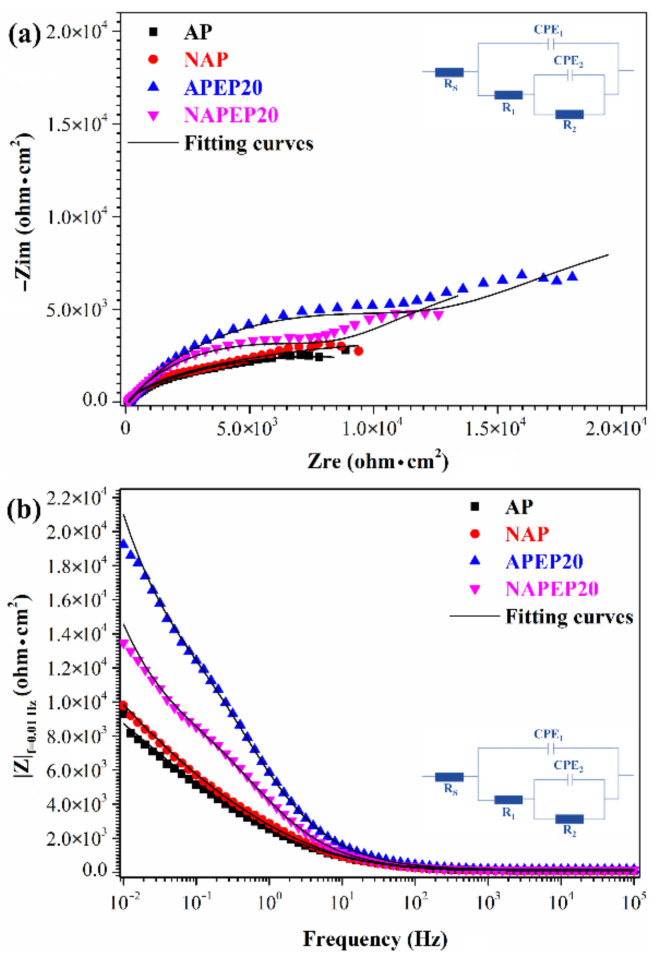
The (**a**) Niquest and (**b**) Bode results for the NAP, AP, NAPEP20 and APEP20 samples in 3.5 wt.% NaCl solution.

Figure 11 shows the Nyquist and Bode results for NAP, AP, NAPEP20 and APEP20 samples in 3.5 wt.% NaCl solution, to verify potentiodynamic polarization measurement results. There are two capacitive arcs at high and low frequencies for all cases. The greater the diameter of the capacitive arc, the greater is the resistance to corrosion [42,43]. The capacitive loop at high frequencies is related to the corrosion products. The diameter of the low-frequency capacitive loop is associated with the resistance to charge transfer.

The equivalent circuit model for this study is shown in Figure 11a,b. *R_s_* is the solution resistance, *R*_1_ is the resistance of the corrosion products, *CPE_1_* is the constant phase element (CPE) for the corrosion product, *R_2_* is the resistance to charge transfer and *CPE_2_* is the CPE for the double layer at the interface between the bottom of the corrosion product and the substrate. ZSimpWin 3.21 is used to fit the test data and the results are listed in Table 2. The values for *R_1_* and *R_2_* are used to characterize resistance to corrosion. The APEP20 specimen is most resistant to corrosion. The curve is similar to the potentiodynamic polarization curve and the impedance of the electropolished samples is greater than that of the sample that is not electropolished. As the duration of electropolishing increases, the corrosion resistance increases but the absolute impedance (*|Z|_f=_*_0.01 Hz_) at low frequency (0.01 Hz) for the NAP sample is greater than that for the AP sample, as shown in Figure 11b. This supports the findings in in the previous section that simple carboxylic acids (OA) increase the reaction rate by dissolving the passive layer on the surface of a Ti-based alloy. The decreased presence of organic ligands is primarily attributed to the elimination of surface passivation layers via chelating of organic ligands with titanium (hydro)oxides, so active sites on the surface of the Ti-based alloy remain exposed.

## 4. Conclusions

A pre-treatment to reduce the longevity of passive layers and increase the efficiency of the electropolishing process is proposed. This study uses oxalic acid to remove the passivation layer from as-fabricated additive manufactured titanium-based materials, so the electropolishing process produces a fine surface roughness.

The XRD results show that NAP, AP, NAPEP20 and APEP20 samples have an as-fabricated substrate crystal structure that is mainly composed of α-Ti phase. A feasible EP system is developed and the surface roughness of the APEP20 sample is reduced from 8.47 µm to 1.09 µm. SEM micrographs show that the surface morphology changes significantly as the duration of electropolishing increases. The surface is initially attached by a large quantity of spherical powder and the surface firstly features waves before becoming smooth. 

Electropolished samples have a higher hardness value than samples that are not electropolished because the surface roughness value is lower. Electrochemical measurements show that the as-fabricated sample has the highest *E_corr_* and *i_corr_* values of all of the test samples. During the electropolishing process, the NAP and AP samples show identical results in that the values for *E_corr_* and *|Z|_f=_*_0.01 Hz_ increase and the *i_corr_* value decreases as surface roughness decreases.

## Figures and Tables

**Table 1:** The names of the samples in this study.

Names	Activation Process	Electropolishing Process	Operation Time (min)
NAP	N/A	N/A	N/A
AP	√	N/A	N/A
NAPEP10	N/A	√	10
NAPEP20	N/A	√	20
APEP10	√	√	10
APEP20	√	√	20

**Table 2:** Corrosion parameters and impedance for the NAP, AP, NAPEP20 and APEP20 samples in 3.5 wt.% NaCl solution.

	AP	NAP	APEP20	NAPEP20
*E_corr_* (V vs. SCE)	−0.107	−0.078	−0.009	−0.033
*i_corr_* (A/cm^2^)	8.66 × 10^−6^	2.42 × 10^−6^	1.25 × 10^−7^	1.18 × 10^−8^
*R_s_* (ohm·cm^2^)	94.3	95.1	114.8	114.5
*CPE*_1_ (s^n^·μohm^−1^·cm^−2^)	74.7	67.8	40.6	53.7
*CPE*_1_ *- n*	0.69	0.69	0.69	0.69
*R*_1_ (ohm·cm^2^)	2422	3293	12,350	8274
*CPE*_2_ (s^n^·μohm^−1^·cm^−2^)	551.2	503.7	491.8	497.2
*CPE*_2_ *- n*	0.64	0.66	0.66	0.67
*R_2_* (ohm·cm^2^)	8672	9826	32,500	24,410

## Data Availability

No new data were created or analyzed in this study.
